# Early Allergen Introduction in Delaware: A Model to Address Healthcare Disparities and Population Healthcare Costs

**DOI:** 10.7759/cureus.108897

**Published:** 2026-05-15

**Authors:** Arjan S Kahlon, Amanrai S Kahlon, Gregory Marcotte

**Affiliations:** 1 Allergy/Immunology, University of Pennsylvania, Philadelphia, USA; 2 Allergy/Immunology, ENT and Allergy of Delaware, Newark, USA

**Keywords:** allergy/immunology, allergy immunotherapy, delaware, early allergen introduction, pediatrics

## Abstract

Food allergies introduce substantial clinical risks to patients and long-term economic burdens for families and health systems. In the past two decades, pediatric allergen introduction guidelines have come full circle, promoting the introduction of allergenic foods during infancy to support immune tolerance and reduce the likelihood of developing lifelong allergies. However, the translation of this clinical evidence into real-world policy and practice remains unevenly distributed; adoption barriers disproportionately affect families facing financial constraints, limited access to consistent guidance, or heightened fear of adverse reactions. If legislated as a preventive measure, early allergen introductions could aim to improve health equity, narrow socioeconomic and racial disparities in community adoption, and reduce downstream expenditures associated with chronic management and emergency care. This narrative review and policy commentary reviews the strong clinical and public policy rationale for early allergen introduction, outlines the equity implications of statewide coverage mandates, and discusses the passage of an innovative policy mandating statewide coverage in Delaware. Comparative emerging efforts in other states and Medicaid programs that could represent subsequent steps in a broader shift toward population-level prevention are also discussed. Ongoing evaluation of patient adherence, food allergy incidence rates, and the policy's cost-effectiveness will be essential to determine whether Delaware’s model can sustainably reduce new food allergy cases and to inform policy nationwide.

## Introduction and background

Across the United States, food allergies impact millions of children, imposing significant health burdens on them and economic burdens on their caretakers. Approximately one in 13 American children has a food allergy, with up to 40% of them having experienced at least one severe, life-threatening allergic reaction, prompting public health efforts to address this increase and to develop campaigns to decrease the allergy rate among newborns [1]. Historically, pediatric guidance from organizations such as the American Academy of Pediatrics (AAP) recommended delaying the introduction of common food allergens in infants to avoid triggering immune reactions that could lead to lifelong allergies. However, a dramatic reversal in the recommendations has occurred, as evidence from prominent trials over the past two decades has emerged, demonstrating that introducing allergenic foods early to infants under 1 year old enhances tolerance development and reduces the risk of food allergy [2]. This evidence has fundamentally reversed the paradigm of avoidance and led to new updated guidelines which encourage early exposure to allergens.

This paradigm shift, through physician and public education, has led to behavioral change: parents no longer routinely avoid early introduction; however, they may not fully implement it for a variety of economic or convenience-related reasons. Thus, policies or guidelines that promote early allergen introduction will increase community-wide adoption and, in turn, improve community health and reduce long-term healthcare costs. On the legislative front, policymakers sometimes adopt these clinical guidelines to advance public health goals, thereby translating these studies into population-level prevention. In 2024, with its House Bill 274, Delaware became the first state in the nation to pass a law mandating insurance coverage of early allergen introduction therapies for infants [3]. By guaranteeing full insurance coverage across both public and private plans, Delaware has ensured that pediatrician-prescribed early allergen introduction (such as peanut or egg products) will be accessible at no cost to families across socioeconomic groups in an effort to address long-term health disparities, ensuring that infants from low-income backgrounds receive equal access to preventative care to thus close the gap in long-term healthcare costs that would be incurred by the development of food allergies [1,4]. Thus, Delaware’s approach serves as a model policy to increase food allergy prevention, improve health equity, and decrease disparities in healthcare expenditures, which disproportionately affect the most cost-sensitive socioeconomic groups. As a narrative review and policy commentary, this article examines the strong clinical and public policy rationale for early allergen introduction.

## Review

Paradigm shift to early allergen introduction

Original Well-Intentioned Guidelines

Pediatric practice in the 1990s and 2000s was guided largely by the now-disproven belief that early allergen exposure might trigger the development of allergies. Parents and pediatricians were advised to delay exposure to allergenic foods, including peanut and egg, until age three, as previously recommended by the AAP in 2000 [5]. By 2008, this advice had begun to be questioned, as observational studies found no benefit of delayed introduction [6]. A true paradigm shift occurred in the mid-2010s, after randomized trials provided definitive evidence that, in direct contrast to the debunked fears of prior years, early introduction of allergens can prevent allergies. This was thought to be achieved by allowing immature immune systems to develop early tolerance rather than exposing mature immune systems to a delayed exposure and, therefore, risk sensitization. The latter may especially be a risk if infants have environmental exposure, rather than ingested exposure, to allergens, such as through the skin, resulting in the immune system’s first exposure to components in a hostile, foreign context rather than a nutritional, digestible one [7].

*Landmark Trials: The Learning Early About Peanut Allergy (LEAP) and *Enquiring About Tolerance (*EAT) Studies*

The most widely recognized of these impactful studies was the LEAP trial, conducted in the United Kingdom, which was a large randomized trial focusing on infants at high risk for peanut allergy. Severe eczema and/or egg allergies, both of which are recognized as immune markers, indicate an elevated risk of developing peanut allergy later [8]. Over 600 high-risk infants were assigned either to consume peanut regularly from infancy onward or to avoid peanut entirely until age five. The results were strikingly clear: the rate of developing a peanut allergy was nearly six times higher in the avoidance condition (17% of infants) than in the exposure condition (3% of infants) by age five. The LEAP study suggested that early avoidance guidelines may have inadvertently contributed to the increase in allergy rates among children. More promisingly, with an over 80% relative reduction in peanut allergy risk among high-risk infants, the LEAP study demonstrated the efficacy of early allergen introduction in preventing peanut allergy [8].

The EAT study followed in the UK shortly after, assigning over 1,300 infants to either an early allergen introduction group exposed to peanut, egg, milk, sesame, fish, and wheat beginning as early as three months of age or a standard introduction group following more traditional feeding practices that did not lead to the introduction of those six allergens until approximately six months of age or later [9,10]. A low adherence to allergen exposure within the early introduction group led to an intention-to-treat analysis showing only a trending but not statistically significant reduction in rates of allergy development compared to the reference group [10], but the per-protocol analysis (including only infants who successfully complied with all aspects of the early introduction diet) demonstrated significant reductions in allergy development rates. No infants (0%) who successfully consumed peanuts regularly in the first year developed a peanut allergy, compared to a 2.5% rate of peanut allergy development in the reference group by age three [10]. Similarly, infants who adhered to the early egg introduction protocol developed egg allergies at a rate of 1.4%, compared with 5.5% in the reference group, representing a nearly 75% relative reduction [10]. With the addition of a larger, unselected sample size and a control group representative of common feeding practices, the EAT study reproduced the LEAP study’s results, showing them to be generalizable and further supporting regular early allergen exposure (Table 1).

**Table 1:** Comparison of LEAP and EAT trials LEAP: Learning Early About Peanut Allergy; EAT: Enquiring About Tolerance

	LEAP study	EAT study
Study population	High-risk infants	General population, breastfed
N	640	~1300
Start age	4-11 months	3 months
Foods assessed	Peanut	Peanut, egg, milk, sesame, fish, wheat
Duration	For 5 years	To 3 years
Main variable	Early peanut intro vs avoid	Early multi-allergen intro vs control
Key outcome	86% reduction in peanut allergy (per-protocol)	Significant reduction in egg and peanut allergy for adherent families (per-protocol) ^[1][2]^

Recent follow-up data from the LEAP-Trio study, published in 2024, confirmed that the protective effect of early peanut introduction persists durably into adolescence. At a median age of 13 years, participants in the original infant peanut-consumption group had a 71% lower prevalence of peanut allergy compared to the avoidance group (4.4% vs. 15.4%), even when consumption patterns varied throughout later childhood [11]. These long-term results strengthen the case for early allergen introduction as a durable, preventive intervention with lasting public health benefit.

Changing Clinical Guidelines

Clinical guidelines have evolved in response to this and other bodies of conclusive evidence supporting early allergen introduction. In 2017, the US National Institute of Allergy and Infectious Diseases (NIAID) issued Addendum Guidelines suggesting peanut exposure as early as four months of age for those at high risk and no later than 6 months of age for those at average or low risk of developing peanut allergies [12,13]; these federal guidelines are in line with the most recent recommendations of academic societies including the AAP and the American Academy of Allergy, Asthma & Immunology [14,15]. The consensus among policymakers, based on randomized trials and resulting clinical guidelines, is clear: once infants are developmentally ready to consume solid food, earlier allergen introduction is preferable to prime infants’ immune systems early to recognize common allergenic foods as harmless. 

Delaware’s House Bill 274: mandating coverage of early introduction supplements

As with all novel clinical developments, especially preventive measures, uptake among the general population is perhaps the most significant hurdle in the development and delivery of a treatment. Such adoption barriers tend to disproportionately affect families from backgrounds lacking resources or guidance, contributing to gaps in health and eventual healthcare expenditure between racial and socioeconomic groups [16]. Passed by the Delaware General Assembly and signed into law by Governor John Carney on August 29, 2024, Delaware’s House Bill 274 is the first state law in the nation to attempt lifelong food allergy prevention on a population scale via early allergen introduction [1,4]. The law requires all health insurance plans offered in Delaware to cover the cost of early allergen introduction dietary supplements for infants, specifically at least one peanut product and one egg product, when prescribed by a healthcare provider [1,4]. Because this mandate extends to programs administered jointly by state and federal entities (including Medicaid and CHIP), this bill allocates state and federal funds for preventive care for low-income families. Because the law requires that early allergen introduction supplements, when prescribed, be available at no out-of-pocket cost, this novel legislation ensures that financial barriers do not prevent any Delaware infant from receiving this preventive care. The stated goals of the legislation include reducing existing socioeconomic and racial disparities in access to care and reducing future state healthcare costs associated with preventable lifelong allergies.

Under HB 274, a pediatric provider (e.g., a pediatrician) may prescribe an early peanut and egg introduction supplement for infants who can tolerate solid foods, typically at a four- to six-month well-child visit. Because the supplements are commonly produced in powder or puree packets that contain the allergenic protein, they can be added to infants’ existing food or formula. Compared with providing whole or home-ground peanuts, for example, this medium allows parents to provide allergen exposure at a measured, consistent level while minimizing the risk of choking or other hazards. Together, these factors promote the safe, predictable, and regular introduction of the allergen over time, as prescribed, minimizing both the acute risk of a reaction and the long-term risk of developing a lifelong allergy. By legislating no-cost insurance coverage, Delaware has integrated effective early allergen introduction into routine preventive pediatric care, similar (but not exactly like) to how vaccines might be prescribed, covered, and administered, rather than leaving it as an optional practice only accessible to those who either can pay out-of-pocket or those independently up to date on medical guidelines and willing to provide their own unsupervised dosing. 

In 2022, the Medical Society of Delaware adopted a resolution, “Addressing Health Disparities in Food Allergy Prevention,” urging equal access to infant-safe peanut and egg proteins. During the 2024 legislative session in Delaware, HB 274 was supported broadly by advocacy organizations and medical groups across pediatrics, public health, and, of course, allergy. Food Allergy Research & Education (FARE), a leading national nonprofit in the space, lauded Delaware for “setting a national example as the first state to address food allergy prevention” [4]. State Representative Kimberly Williams, the bill’s sponsor, notes that the bill “has the power to change so many lives… With Delaware investing early on, this will enable individuals … [without] parents hav[ing] to worry each and every day about the health risks their child takes every time they walk out the door” [4]. 

Addressing health disparities through an early introduction policy

A key motivator behind Delaware’s HB 274 is advancing health equity. Despite its established status as a standard of care, early allergen introduction is not uniformly adopted by families; there are significant disparities across racial and socioeconomic groups in the timing of allergen introduction. 

Parents of Black children reported having introduced peanut, egg, and milk to their children later than parents of white children [17]. These significant gaps in adherence to early feeding guidelines may be a primary factor underlying significantly higher allergy rates for Black and Hispanic children compared to White children, indicating that some communities bear disproportionate burdens of the outcome (lifelong food allergies) that early allergen introduction can mitigate [18,19]. Beyond these racial disparities, additional socioeconomic barriers to early allergen introduction persist: constrained by cost or availability, families with limited resources may struggle to provide consistent early exposure to allergens, especially when guidance from a healthcare professional is limited or when there is fear of dangerous and costly allergic reactions [16]. Commercially available early allergen introduction products offer greater consistency and convenience, potentially reducing the risk of reactions, but can cost up to a few hundred dollars per preventive treatment course, making them financially out of reach for many families.

Recent real-world evidence from a multistate pediatric primary care network (treating across disparate settings of populations, geography, states, and insurances) demonstrated significant declines in peanut and overall IgE-mediated food allergy diagnosis rates following the publication of the 2015 and 2017 early introduction guidelines [20]. This study, including the regional network of the Children’s Hospital of Philadelphia with multiple sites in Delaware, supports the population-level impact of these recommendations and reinforces the case for policies like HB 274 that operationalize guideline uptake [20].

By mandating full insurance coverage by Medicaid of early allergen introduction supplements, Delaware’s HB 274 directly addresses these disparities, ensuring that all families across socioeconomic groups and insurance plans have the same, guaranteed, low-barrier access to early allergen exposure, a critical component, as public health campaigns often fail to reach those most at risk due to the costs of treatment and adherence, even if low. As the costs associated with food allergies, from special diets and medical care to lost opportunities for children and lost wages for parents, often “harm those who can least afford it,” lowering allergy development rates in already disadvantaged populations would yield outsized returns to narrow the racial and economic gaps in allergy prevalence [4].

Delaware’s law also empowers pediatric providers in disadvantaged communities to treat their patients more effectively. HB 274 thus operationalizes an otherwise abstract academic guideline (exposure to allergens early and consistently) by providing a clear, trustworthy, low-barrier preventive treatment regimen. The State’s goal is to both improve health outcomes and lower state and federal costs of long-term care for these patients. 

It is notable that self-insured plans (Employee Retirement Income Security Act (ERISA) plans) are exempt from state regulations. However, by mandating this for the entire community, the legislation also creates a unified community approach and standard of care. This community standard of care may increase behavioral change across insurance coverage groups.

Population health and cost implications

Beyond their medical impacts, food allergies impose significant economic burdens on affected families and represent a resource cost for the healthcare system as a whole. In addition to daily maintenance costs for allergen-free food, families must pay for acute, life-saving epinephrine autoinjectors such as the EpiPen, as well as emergency medical transport, services, and potentially hospitalization in the event of severe reactions. The annual total economic cost, including medical costs and other expenses, of food allergies in children across the US was estimated at nearly $25 billion in 2013, a figure that has likely increased with rising population and allergen prevalence in the past dozen years [21,22]. FARE estimated that each food-allergic child costs families an additional $4,200 per year in direct and indirect costs at the same time, with peanut allergies being the costliest at a medical and opportunity cost of over $7,200 per year per child, bringing total peanut allergy costs for each family throughout childhood to over $130,000 per child [21,23].

The Federal ERISA preemption of state laws effectively limits state efforts to control healthcare costs and translate clinical guidelines into healthcare policy by exempting self-insured employer-sponsored coverage (which comprises 64% of employer-sponsored coverage) [24]. However, the remaining fully insured employer-sponsored insurance plans, the state employees' benefits plan, and state Medicaid enrollees would be covered by the new state mandate, and as these three groups comprise approximately 50% of the state’s covered lives, there remains a significant opportunity for long-term healthcare savings [25]. For example, with Medicaid covering 43% of the 11,000 births each year in Delaware, this policy will cover (at a minimum) 4,700 newborns annually through Medicaid alone, representing up to $19.74 million in cost savings per year for families prescribed early allergen introduction products [26]. As noted earlier, actual long-term healthcare savings could rise even higher when accounting specifically for peanut allergy costs averted over patients' lifetimes.

In addition to direct medical costs, reducing the prevalence of food allergies among children would yield broader population health benefits that may be harder to quantify but remain significant. Children without food allergies experience fewer restrictions on their activities and diets and thus may be less likely to develop anxiety in social situations. Their parents may experience less emotional stress, resulting in an overall improved quality of life for allergy-free families. Lower allergy prevalence would also reduce the strain on childcare providers, such as teachers, to accommodate allergic children through special plans, training, and allergy zones. While these factors may be absent from cost-effectiveness analyses of the intervention, these intangible benefits, allowing Delaware children to, on average, lead healthier, more carefree lives, undoubtedly contribute to the value of the intervention.

Preventing even a portion of infants from developing lifelong food allergies via early allergen introduction would thus potentially yield significant cost savings over the course of childhood and beyond, on the order of tens of millions when extrapolated to a state population, with savings both for individual families and government agencies responsible for the healthcare insurance and costs of individuals enrolled in programs such as Medicaid. As families receive access to early allergen introduction products in Delaware, rigorous cost-effectiveness studies are needed to estimate the overall cost savings when factoring in prescription and adherence rates, and could provide real-world data on not only those metrics but also changes in allergy outcomes in the insured population to enable long-term analyses of cost effectiveness in an environment of rising medical costs for treatment.

Broader impact and emerging initiatives in other states

The Delaware model for early allergen introduction policy may serve as a framework for other jurisdictions to implement their own legislation at the state, insurer, or federal levels. In New York (Senate Bill S7915 and Assembly counterpart A812) and neighboring Pennsylvania (House Bill 672), legislators are mirroring the Delaware approach by requiring state insurance plans to cover early peanut and egg introduction supplements for infants at no cost to families [27,28]. Kentucky has taken a unique approach to make early allergen introduction accessible without waiting for the legislative process. Through managed care plans, including WellCare of Kentucky, the state’s Medicaid program launched a 2025 initiative to provide free early allergen introduction kits to enrolled infants, with powder mixes covering peanuts, eggs, and milk [29]. This unique approach represents an instance of Medicaid programs innovating in patient care through plan benefits and also provides an alternative model that operates outside the legislative system. With interest in the adoption of policies promoting early allergen introduction growing in states across the nation, federal intervention should also be considered. The Children’s Hospital of Philadelphia has urged the USDA’s WIC program to incorporate early allergen foods, so that WIC participants receive exposure to common allergens by four to six months of age [30].

Thus far, the aforementioned legislative and governmental efforts have been bipartisan, backed primarily by advocacy organizations (such as FARE and the Allergy & Asthma Network) and medical organizations (such as the Medical Society of Delaware and the AAP), which have been vocal in encouraging other states to “follow Delaware’s lead” [1]. As these efforts advance, their adoption could broaden the scale of guaranteed early allergen introduction and validate the policy’s feasibility in populations and health systems larger than Delaware. If the Delaware policy model shows promising results in producing measurable declines in new food allergy cases, the state may well catalyze a nationwide uptake of early allergen introduction as a standard covered benefit. 

Limitations of legislation and regulation in healthcare policy innovation

Several limitations to this new health policy (and all similar government healthcare mandates) do exist. This model in Delaware represents an innovative policy model based on strong clinical prevention evidence, but the real-world impact of mandated coverage on adherence, incidence, equity, and cost remains uncertain and requires formal evaluation. For example, the LEAP and EAT studies do show clinical efficacy and outcome, but do not directly establish that mandating insurance coverage for commercial early allergen introduction products will produce similar adherence, effectiveness, equity gains, or cost savings at the population level. While existing estimates of the economic burden of food allergies on Delawareans and Americans more broadly are helpful for contextualizing the potential impact of HB274, they do not provide specific evidence on the extent to which the policy will be cost-saving. The projections provided are currently hypothetical and contingent on clinician access, treatment uptake, treatment adherence, and other factors determining real-world effectiveness. Further, the elimination of cost barriers for insured families to access early allergen introduction supplements once prescribed does not address other barriers, including clinician access, parental trust, cultural feeding practices, healthcare literacy, and implementation support. As racial and socioeconomic inequities in health and healthcare are a product of these and other factors, legislating free insurance coverage may not alone eliminate inequities in adoption or outcomes unless steps are taken to ensure that families in need are, in fact, accessing the intervention highlighted [31]. For example, coverage mandates differ between federal (Medicare) and state (Medicaid or state employee) plans, and between commercial ERISA fully-insured and self-insured plans. Government legislation and regulations, therefore, differ in implementation across these varieties of health insurance coverages. Despite this health policy's intent, the uninsured still have no coverage and remain at risk. Therefore, this innovative legislation in Delaware represents a promising policy experiment, the clinical and population health effectiveness of which remains to be evaluated prospectively.

## Conclusions

The passage of Delaware’s HB 274 marks a major milestone in the effort to address rising food allergy rates among children. By mandating insurance coverage for early introduction of peanut and egg for infants, Delaware has taken action to translate modern medical guidelines into practical preventive care accessible to all segments of the population, thereby addressing both the incidence of the disease and the inequities that arise from its unequal distribution and delayed medical treatment. While implementation may still pose a barrier, especially among medically distrustful families or those with limited access to healthcare providers, Delaware’s approach arguably removes the largest barrier, demonstrating how health policy can translate clinical research into population-wide improvements in health. By ensuring that prevention is not a luxury good but a standard benefit accessible to every infant, Delaware’s initiative promotes foreground health equity to address well-documented disparities in both allergy prevalence and access to care. If successful, the Delaware policy may reduce the volume of childhood food allergies and the associated pains for individuals and families, and also possibly yield long-term savings in health expenditures, for the very insurance payors affected by HB 274, by averting the cost of treating a large proportion of patients who might otherwise develop food allergies as a chronic condition requiring lifelong management and care. If a similar policy were implemented nationwide, even modest reductions in food allergy prevalence could provide healthcare savings beyond the quality-of-life improvements for children. 

Delaware’s pioneering efforts in preventive pediatric health policy have laid the groundwork for similar legislative advances in other states. As similar efforts unfold across the nation, it will be crucial to track metrics including the uptake of and adherence to prescribed treatment, later reductions in new allergy diagnoses, and the overall costs and benefits afforded by the program, starting with the early launch of Delaware’s initiative, which will inform future policy decisions. Achieving accessible early allergen introduction as a standard preventive practice nationwide, like immunizations, to reduce the incidence of food allergies in future generations will require evaluations of statewide programs. Delaware’s action on HB 274 thus marks a new chapter in food allergy care, focused not only on early intervention but also on health equity to support the long-term well-being of children across the population.
